# Does Early Surgical Treatment in Degenerative Cervical Myelopathy Have a Favorable Clinical Outcome and Impact on Quality of Life?

**DOI:** 10.3390/jcm15124844

**Published:** 2026-06-22

**Authors:** Michele Incerti, Paola M. F. Cristaldi, Andrea Parlangeli, Vittorio Ricciuti, Federica Balletti, Daniele Nicoli, Clarissa Cavadoli, Franco Servadei

**Affiliations:** 1 Unit of Neurosurgery, Policlinico di Monza, 20900 Monza, Italy; 2 Department of Medicine and Surgery, School of Medicine, University of Milano-Bicocca, 20126 Milan, Italy; 3 Global Neurosurgery Programme, Fondazione IRCCS Istituto Neurologico Carlo Besta, 20133 Milan, Italy

**Keywords:** mild degenerative cervical myelopathy, mild DCM, mJOA, quality of life, early surgery, surgical outcomes

## Abstract

**Background/Objectives:** degenerative cervical myelopathy (DCM) is the leading cause of spinal cord impairment in adults, often resulting in disability and reduced quality of life (QoL). Surgery is recommended for moderate and severe cases, while its role in mild DCM remains debated. Emerging evidence suggests that early surgery may improve outcomes, particularly QoL. **Methods:** We conducted a retrospective, single-center observational study of a cohort of patients undergoing cervical spine surgery for DCM between January 2020 and August 2023 at a single institution (Policlinico di Monza, Italy). Demographic, clinical, radiological and surgical data, as well as complications and outcomes, were analyzed. Neurological status was assessed using the modified Japanese Orthopedic Association (mJOA) score and QoL was evaluated using the Short Form-36 (SF-36) questionnaire preoperatively, at discharge, and at follow-up. **Results:** 51 patients were included (mean age 58.1 years; 41% female), with anterior surgery performed in 67%. Mild preoperative mJOA score was observed in 74% of patients. At follow-up, 65% achieved complete recovery, 29% improved, and 6% remained stable. No neurological deterioration was recorded. Univariate analysis identified age, anterior cervical discectomy and fusion (ACDF), and mild preoperative mJOA score as significant predictors of recovery. Multivariate logistic regression analysis identified mild preoperative mJOA score as a strong independent predictor of complete clinical recovery (OR = 240.64, 95% CI: 6.82–8496.22, *p* = 0.002). SF-36 showed significant improvements in emotional well-being, social functioning, pain, and general health, particularly in mild cases. Complications were low (5.8%) and limited to transient dysphagia. **Conclusions**: early surgical treatment in selected patients with mild DCM may be associated with favorable neurological and quality-of-life outcomes, although larger prospective studies are needed.

## 1. Introduction

Degenerative cervical myelopathy (DCM) is the most common cause of non-traumatic spinal cord impairment in adults, resulting from compression of the spinal cord secondary to the progressive degeneration of cervical osseous and soft tissue structural components [1]. Radiological evidence of cervical spinal cord compression (SCC) is estimated to affect up to about 24% of healthy adults, and prevalence is expected to rise with an aging population [2]. While most people with SCC remain asymptomatic, a proportion (about 2 to 4%) develop symptoms of DCM [2]. Clinical manifestations exist on a spectrum of severity: while some patients may present with minor signs and symptoms and have a relatively good quality of life, severely affected patients may be unable to walk and even quadriplegic. The natural history of DCM shows a high variability. While some patients experience a slow and stepwise deterioration, others experience prolonged periods of stability [3,4]. However, abrupt worsening can occur, often but not exclusively following minor neck injuries [5,6,7]. The diagnosis of DCM requires clinical signs and symptoms of cervical myelopathy and imaging evidence of cervical cord compression [8]. Magnetic resonance imaging (MRI) is the imaging modality of choice in the case of suspected DCM. Increased T2 signal is sometimes seen in the spinal cord, suggesting spinal cord damage. Computed tomography (CT) may be used when MRI is contraindicated or for better delineation of bony elements, including osteophytes, facet joint hypertrophy, and ossification of the posterior longitudinal ligament (OPLL), which may contribute to spinal cord compression [9]. Weight-bearing films, including flexion-extension radiographs, are the study for the assessment of cervical lordosis and evidence of instability that may predispose to a repetitive trauma mechanism underlying DCM [10,11]. The modified Japanese Orthopedic Association (mJOA) score [12,13] (Table 1) quantifies the severity of myelopathy. Mild DCM can be defined as an mJOA score from 15 to 17, moderate as an mJOA score from 12 to 14 and severe as an mJOA score from 0 to 11. According to the AOSpine clinical practice guidelines [14], patients with severe and moderate DCM should be referred for surgery unless contraindicated, although it remains unclear whether patients with mild DCM will benefit from surgery when compared to conservative management. In other words, there are no strong recommendations for surgery in patients with mild DCM, although some authors suggest that early surgery in this population may give better results in terms of quality of life, compared to a wait-and-see strategy that may lead to neurological deterioration [15]. Given the unpredictable outcomes of conservative management in halting progressive deficits, current clinical trends increasingly favor early surgery to prevent irreversible spinal cord damage. Consequently, modern spine care emphasizes shared decision-making to balance the risks of mild progression against the benefits of timely decompression. Additionally, Enhanced Recovery After Surgery (ERAS) pathways have optimized perioperative care, reducing morbidity and accelerating functional recovery.

Although the mJOA scale remains the gold standard for assessing objective neurological impairment in DCM, it primarily captures motor and sensory deficits while often underestimating subtler limitations in daily living, social roles, and mental well-being. Therefore, prioritizing Quality of Life (QoL) outcomes—such as those evaluated by the SF-36 questionnaire—alongside mJOA recovery is essential to capture the true, comprehensive impact of the disease and its surgical treatment from the patient’s perspective, particularly in mild presentations.

The main aim of this manuscript is to evaluate whether patients with mild DCM, as defined by the mJOA, benefit from early surgical treatment in terms of clinical outcomes and quality of life (QoL), testing the explicit null hypothesis (H0) that early surgery offers no significant difference in clinical recovery rates or post-operative QoL parameters. Secondarily, we aim to determine whether the rate of perioperative complications justifies the surgical approach in patients with minimal clinical symptoms.

## 2. Materials and Methods

**Figure 1:**
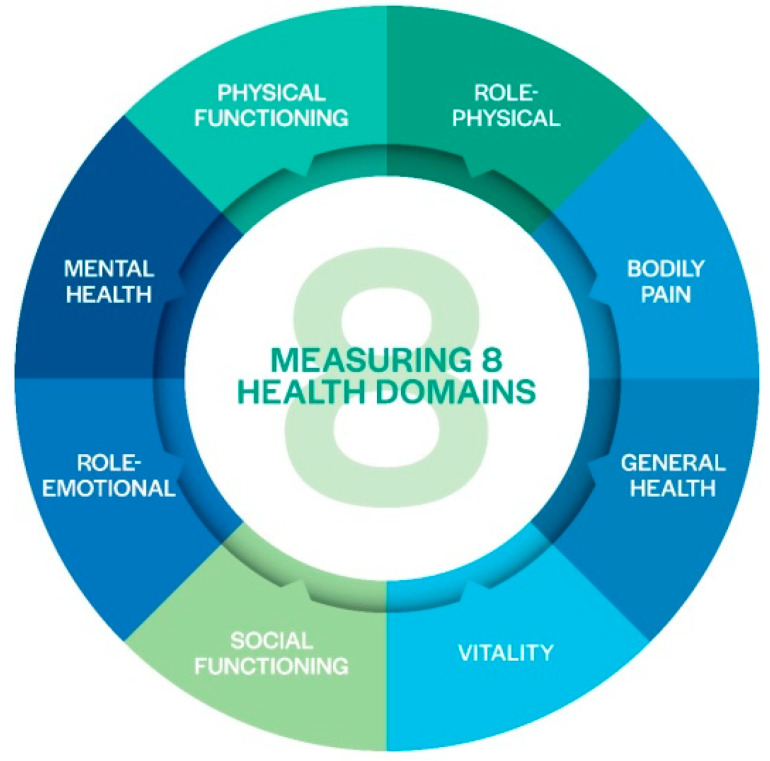
Short Form Health Survey 36 variables (SF-36).

This retrospective study includes patients who underwent cervical spine surgery for DCM between January 2020 and August 2023 at a single institution (Policlinico di Monza, Monza, Italy). Criteria for inclusion in the study included those patients who had undergone cervical spine surgery (one-, two-, or three levels, instrumented or non-instrumented fusion, anterior and/or posterior approaches) with a preoperative diagnosis of DCM. All surgeries were performed by a single surgeon (MI). A diagnosis of DCM was established based on the presence of at least one clinical sign of myelopathy, such as hypereflexia or gait disturbance, and MRI-documented cervical spinal cord compression with medullar hyperintensity on T2 sequences. We followed STROBE guidelines for reporting [16]. Medical records were prospectively collected and retrospectively reviewed for demographic information (sex, age at surgery, year of surgery), preoperative clinical presentation, symptom duration, radiologic preoperative findings, cervical instability defined by the presence of vertebral hypermobility at the morphodynamical cervical x-rays, surgical approach, peri- and postoperative complications, outcomes, and neurologic examination from the immediate postoperative period to the discharge period and follow-up periods (at 1, 3 and 6 months). Neurologic conditions before and after surgery were assessed using the mJOA score, evaluated both preoperatively and at least at six months postoperatively. Patients were then divided into two groups: complete recovery, for those who reached a postoperative mJOA score of 18/18, and non-complete recovery for those who had partial clinical improvement or clinical stability after surgery. QoL was evaluated using the Short Form Health Survey 36 (SF-36) score (Figure 1), administered through patient interviews preoperatively and at the six-month follow-up. To minimize interviewer and response bias, postoperative SF-36 health status telephone interviews were administered by trained clinical research assistants who were not part of the direct surgical treating team. However, because this registry only captures patients managed surgically, individuals with mild DCM who were treated conservatively were not evaluated, introducing a potential selection bias that limits direct comparison between operative and non-operative strategies. “Early surgery” was defined conceptually based on the clinical stage of neurological impairment at presentation rather than an arbitrary symptom duration threshold, specifically referring to decompression performed while patients were still in a mild stage of disease.

Statistical analysis was performed using IBM SPSS Version 29.

Continuous variables are presented as mean ± standard deviation or median (interquartile range), as appropriate, whereas categorical variables are reported as frequencies and percentages. Associations between categorical variables were assessed using the Chi-square test or Fisher’s exact test when appropriate. Comparisons involving ordinal variables, including mJOA categories, were performed using non-parametric tests. Logistic regression analysis was used to identify predictors of complete recovery. Odds ratios (ORs) are reported together with their 95% confidence intervals (95% CI). Because eight SF-36 domains were analyzed, correction for multiple comparisons was applied using the Bonferroni method. Effect sizes and 95% confidence intervals are reported whenever appropriate. A two-sided *p*-value < 0.05 was considered statistically significant.

## 3. Results

Patients with missing primary outcome variables or incomplete follow-up clinical scores were managed using a complete-case approach (listwise deletion). Consequently, 4 patients out of the 55 initially screened were excluded due to incomplete postoperative data, resulting in a final analytical cohort of 51 patients (41% female, mean age 58.1 years). Preoperative MRI showed nearly equal distribution between single- and multi-level cord compression. Most patients (67%) underwent anterior surgery. Preoperative mJOA scores were mostly mild (74%). Postoperatively, 65% achieved complete recovery, 29% improved, and 6% remained stable. No patients experienced a worsening of neurological conditions. The mean symptom duration prior to operative intervention was 12.4 ± 6.1 months (range: 2–31 months).

Patients’ characteristics are listed in Table 2.

**Figure 2:**
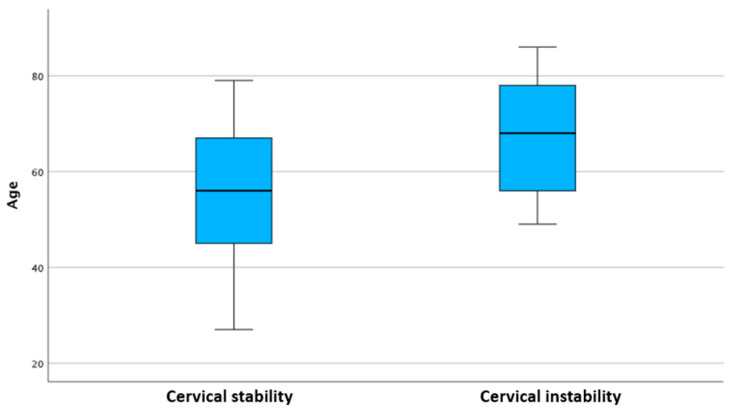
Correlation between age and cervical stability.

**Figure 3:**
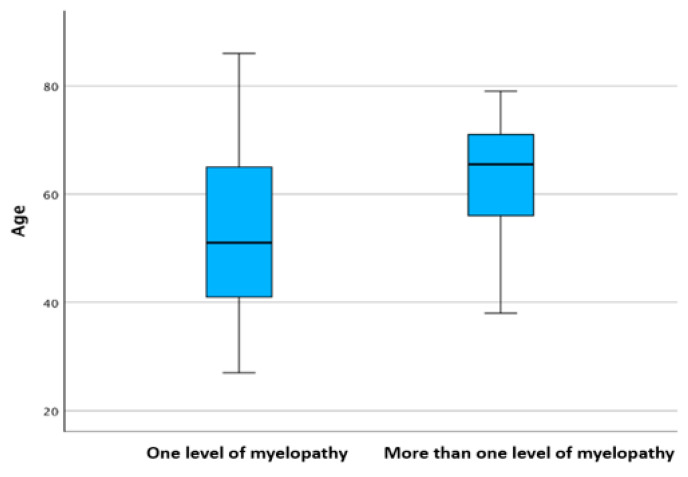
Correlation between age and levels of myelopathy.

**Figure 4:**
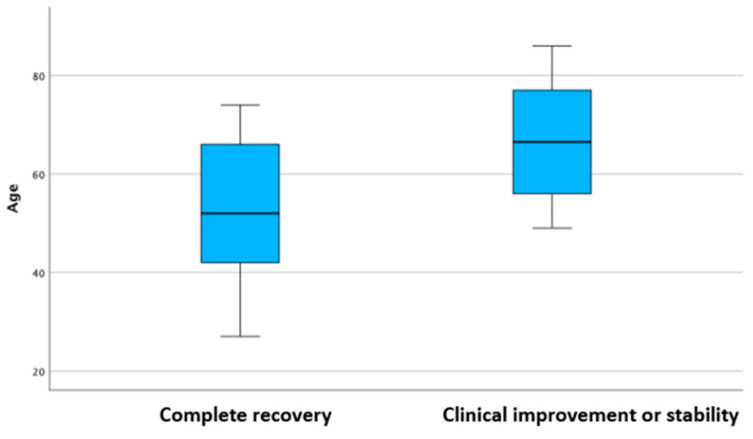
Correlation between age and recovery.

The *t*-test found a statistically significant correlation between age and cervical instability (*p* = 0.021), age and levels of myelopathy involved (*p* = 0.003), and age and complete recovery (*p* < 0.001). Results are illustrated in the following box plots (Figure 2, Figure 3 and Figure 4).

**Figure 5:**
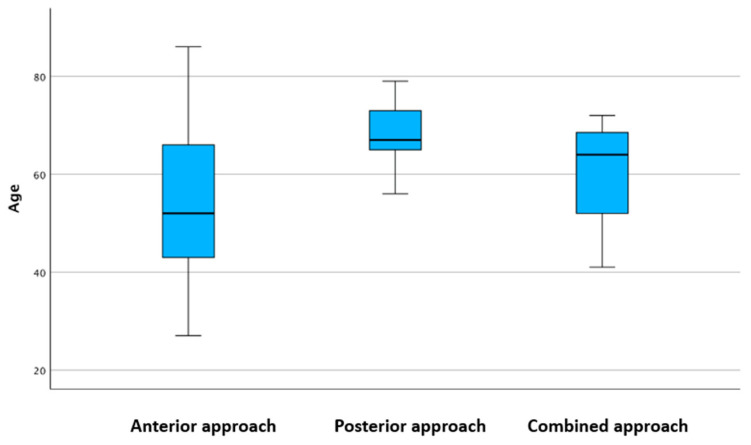
Correlation between age and diagnosis and age and surgical approach.

**Figure 6:**
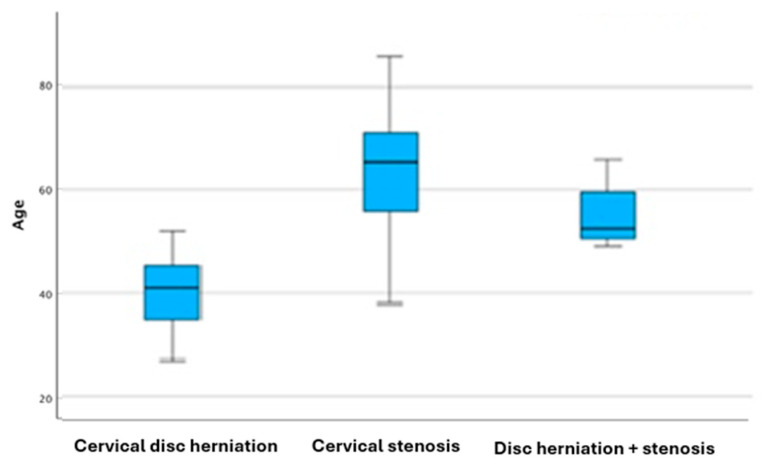
Correlation between age and surgical approach.

**Figure 7:**
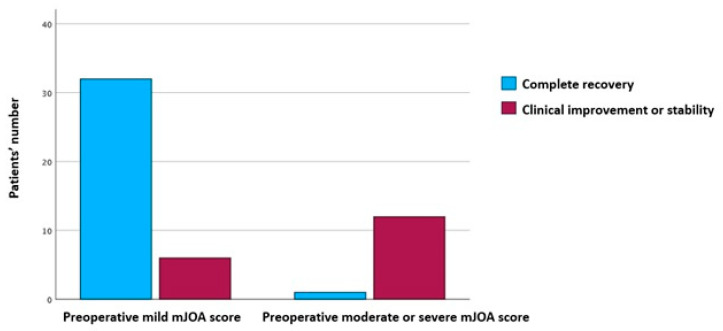
Correlation between preoperative mJOA score and recovery.

ANOVA tests also found a statistically significant association between age and diagnosis (*p* < 0.001) and age and surgical approach (*p* = 0.007). Results are illustrated in the following box plots (Figure 5 and Figure 6). Univariate analyses (Chi-square test) showed a statistically significant correlation between complete recovery and age, ACDF and preoperative mild mJOA score (Table 3, Figure 7). A multivariate logistic regression was performed to assess the impact of these variables (age, preoperative mild mJOA score and ACDF) on recovery. As shown in Table 4, the strongest predictor of complete recovery was a preoperative mild mJOA score (OR = 240.64, 95% CI: 6.82–8496.22, *p* = 0.002). The wide confidence interval observed for this subgroup is attributable to quasi-complete data separation constraints inherent to the small sample size, as nearly all patients with mild baseline impairment achieved a favorable outcome.

The loss-to-follow-up rate was 7.2%. A comparative analysis between the 4 excluded patients and the 51 included individuals revealed no statistically significant differences in baseline demographics, age, or preoperative neurological status (*p* > 0.05), suggesting that data were missing completely at random. Statistical analysis demonstrated that preoperative symptom duration did not significantly influence final functional or neurological outcomes (*p* > 0.05).

**Figure 8:**
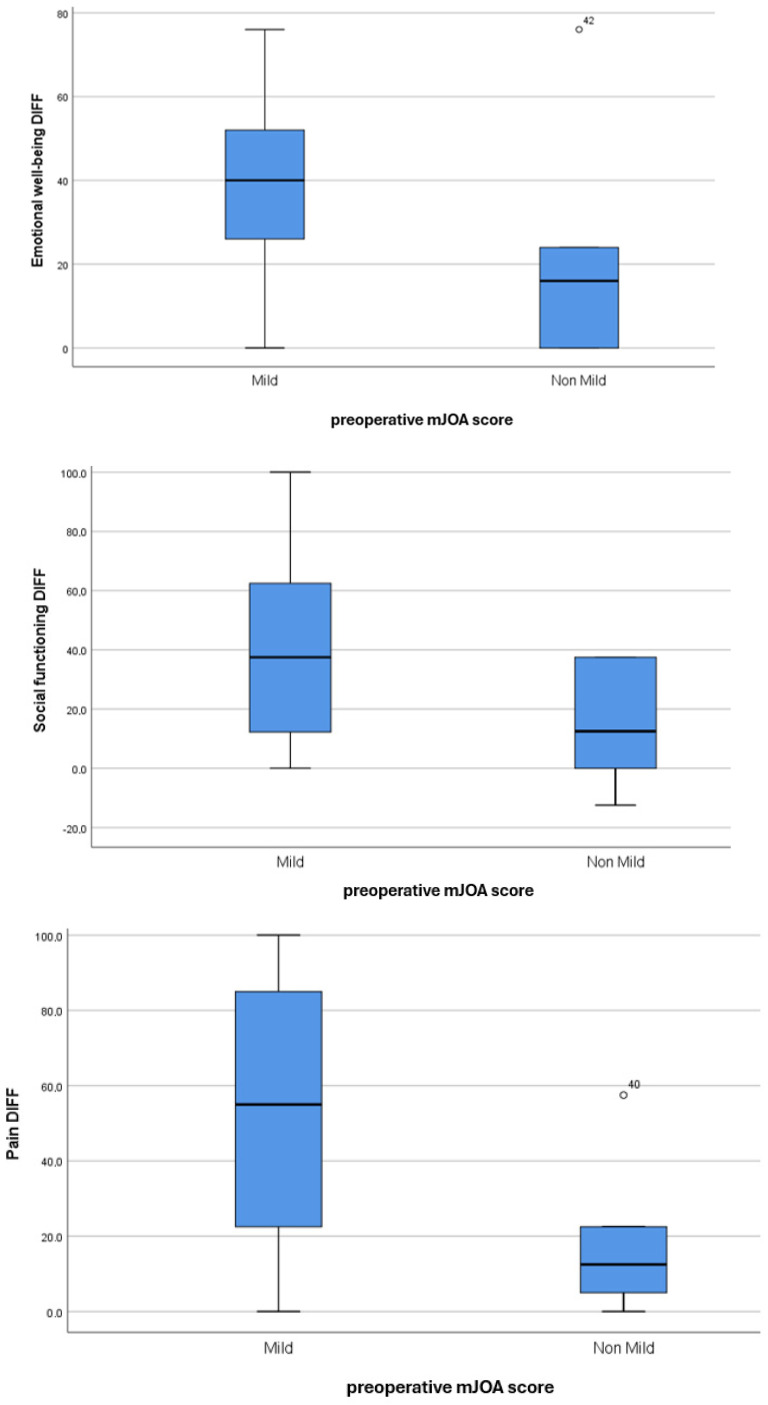

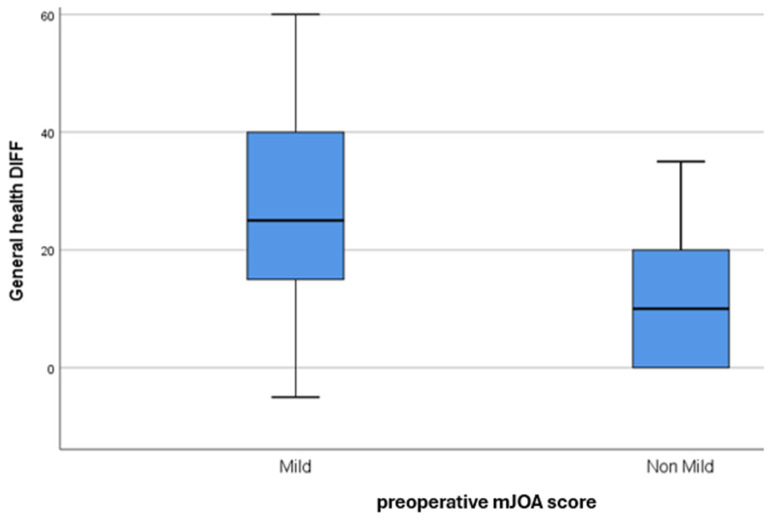
Difference in values of four items of the SF-36 calculated before and after surgery between mild- and non-mild mJOA score patients.

Mann–Whitney test was conducted to compare the two groups (mild mJOA score versus non-mild mJOA score patients) with the items of the SF-36, pre- and postoperatively, showing a statistically significant difference in four of them (emotional well-being, social functioning, pain and general health). The results are illustrated in the following box plots (Figure 8). After applying the Bonferroni correction for multiple comparisons (adjusted threshold *p* < 0.00625), early surgical treatment in mild DCM patients still demonstrated a statistically significant superior improvement in general health (*p* = 0.0018, Cohen’s d = 0.875) and social functioning (*p* = 0.0032, Cohen’s d = 0.824) compared to the moderate/severe group. No statistical differences were noted in the remaining four items (physical functioning, role limitation due to physical health, role limitation due to emotional problems and energy/fatigue).

Finally, our series showed three cases (3/51—5.8%) of transient mild postoperative dysphagia, presented immediately after surgery, and resolved spontaneously within 2 to 4 weeks. Management was strictly conservative, consisting of clinical monitoring and temporary dietary modification. No major or long-lasting complication was noted.

## 4. Discussion

The natural history of DCM is highly variable, but the disease generally progresses over time, particularly in patients who already experience impaired QoL [14,17]. In this study, mild preoperative mJOA score was the strongest predictor of complete recovery, in line with previous studies highlighting the importance of baseline neurological status [18,19,20,21]. Age and the anterior cervical discectomy and fusion (ACDF) approach showed positive trends but did not reach statistical significance. Younger patients may experience better surgical outcomes, as supported by previous literature [22,23,24]. Similarly, ACDF showed a strong trend toward complete recovery, although not statistically significant. Some data support ACDF as an independent predictor of early discharge, but findings remain mixed [23,24,25].

Early surgical intervention is critical, as prolonged spinal cord compression can cause irreversible damage, making early diagnosis and prompt surgical management essential to minimize the risk of permanent disability [26]. Evidence suggests that patients undergoing surgery within four months of symptom onset achieve significantly better long-term mJOA outcomes compared to those treated later [27]. The management of patients with mild DCM remains controversial. Current guidelines do not systematically recommend surgical intervention for patients with mild DCM [14]. Despite this, nonoperative management for mild DCM is frequently ineffective in halting disease progression. Systematic reviews report that 23–54% of initially nonoperatively managed patients eventually require surgery due to clinical worsening within 2 to 6 years [19]. Similarly, one longitudinal cohort study documented neurological deterioration in 50% of mild DCM patients over a 30-month period [28]. Based on these findings, several studies have recommended early surgical intervention even for mild DCM [29,30] to prevent irreversible deterioration, especially considering the improved safety profile of contemporary surgical techniques [14]. While our study lacks a direct conservative control arm to confirm this trend locally, the high rate of complete recovery seen in our early surgical cohort (65%) provides contrasting observational data that supports further investigation into early intervention.

Our results indicate that patients with mild preoperative mJOA scores are more likely to achieve complete recovery and QoL improvements, aligning with existing literature [19,31], likely because their baseline neurological status allows a higher potential for full recovery. QoL improvements measured by SF-36 were more pronounced in mild DCM patients compared to those with moderate or severe disease, marking a novel contribution to the field. Furthermore, our results suggest that even patients with mild DCM experience a substantial impairment in QoL preoperatively. These patients are also the most likely to achieve full recovery and show superior long-term outcomes—particularly if they are younger (Table 2). Notably, the assessment of patient-reported outcomes like the SF-36 highlights that functional recovery is a multidimensional process that extends far beyond objective motor and sensory neurological parameters. Chronic compressive pathologies of the spine often trigger broader systemic implications, including altered pain sensitization and sleep disturbances, which are well-documented key determinants of overall quality of life and patient-centered recovery in general musculoskeletal and osteoarticular disorders [32,33]. Specifically, persistent discomfort can profoundly impact emotional well-being, social functioning, and vitality. By achieving early surgical decompression in mild DCM, we not only protect the spinal cord from irreversible damage but also effectively interrupt the chronic pain cascade, thereby facilitating a comprehensive restoration of the patient’s health-related quality of life. This study appears to be one of the first to directly compare QoL outcomes between mild and non-mild DCM patients in both pre- and postoperative phases, highlighting the importance of incorporating QoL measures alongside traditional motor and sensory scores in clinical assessment and surgical planning [34].

Based on these preliminary findings, our data suggest that early surgical intervention might be a viable option in mild DCM, potentially facilitating a quicker return to work and social activities, thereby reducing the broader societal burden. However, given our study’s lack of a conservative control group and limited cohort, these observations should be interpreted with caution and serve to complement, rather than dictate, clinical judgment. From a surgical standpoint, both anterior and posterior approaches for DCM treatment exhibit low complication rates, with neurological complications reported between 0.9% and 6% [35,36], and major spinal cord injury risk below 0.5% [37,38]. Anterior procedures carry risks such as transient dysphagia, recurrent laryngeal nerve palsy, and esophageal injury, while posterior approaches are more often associated with C5 palsy and axial neck pain [38]. In our series, the postoperative complication rate was 5.8%, limited to transient dysphagia without lasting effects, further supporting the safety of current surgical management.

In conclusion, our results support early surgical intervention for patients with radiological spinal cord compression accompanied by clinical myelopathy signs, especially in younger individuals, to optimize neurological recovery and QoL while maintaining an acceptable risk profile. For asymptomatic patients with spinal cord compression but without MRI T2 hyperintensity or clinical myelopathy, careful monitoring through annual imaging and neurological evaluation remains prudent to detect disease progression promptly. Although our study reinforces the growing consensus favoring surgery in mild DCM, the statistical magnitude of our predictors must be considered preliminary, highlighting the absolute need for larger prospective studies to validate these findings.

## 5. Limitations

Despite its clinical relevance, several limitations of this study must be acknowledged.

First, regarding the study design, its retrospective nature, single-center framework, and single-surgeon experience inherently restrict the generalizability of our findings and lack external validation. Moreover, the small sample size (*n*= 51) introduces specific statistical constraints. Specifically, the limited cohort size leads to a sparse data bias and a potential overfitting in the multivariable logistic regression analysis. This resulted in model instability and an unusually large odds ratio for the mild mJOA score subgroup due to quasi-complete data separation. Furthermore, a potential selection bias is inherent, as patients chosen for surgery represent a highly filtered subgroup judged clinically more likely to benefit based on the surgeon’s experience and specific clinical judgment. Potential temporal bias cannot be entirely excluded either; although procedures were performed by a single surgeon, perioperative management may have subtly evolved between January 2020 and August 2023.

Second, defining “complete recovery” as a post-operative mJOA score of 18/18 introduces possible ceiling effects in mild DCM patients, which inherently favors patients entering the study with higher baseline scores. However, the concurrent and robust improvements in the SF-36 domains confirm that their recovery was clinically meaningful.

Third, the cohort exhibits clinical heterogeneity due to heterogeneous surgical strategies, varying pathology types, and different numbers of operated spinal levels. While this reflects real-world clinical practice, it prevents isolating the effect of a single surgical technique.

Fourth, the study is limited by a relatively short follow-up duration for certain patients; although the mean follow-up was 16.3 months, the minimum duration was 6 months. Longer-term monitoring is essential since neurological and quality-of-life adjustments can evolve over an extended period.

Finally, the absence of a concurrent nonoperative or conservative-treatment comparator group means that definitive conclusions regarding the superiority of early surgery over observation cannot be made.

Therefore, these findings should be considered preliminary, and future large-scale, prospective, multicenter studies incorporating a conservative management arm are mandatory to establish definitive guidelines.

## 6. Conclusions

Our analysis highlights that preoperative mild DCM status is the strongest predictor of complete neurological recovery following surgery, underscoring the potential importance of early diagnosis and timely intervention. Considering the relatively low risks of surgery and the favorable impact on QoL, our findings suggest that early surgery in selected patients with mild DCM may be beneficial, though larger studies are needed. Such future prospective studies with larger cohorts remain mandatory to better clarify the definitive role of surgery in this specific population.

## Figures and Tables

**Table 1:** The mJOA scale.

Category	Score	Description
**Upper extremity motor subscore (/5)**	0	Unable to move hands
	1	Unable to eat with a spoon but able to move hands
	2	Unable to button a shirt but able to eat with a spoon
	3	Able to button a shirt with great difficulty
	4	Able to button a shirt with mild difficulty OR other mild fine motor dysfunction
	5	Normal hand coordination
**Lower extremity score (/7)**	0	Complete loss of movement and sensation
	1	Complete loss of movement, some sensation present
	2	Inability to walk but some movement
	3	Able to walk on flat ground with walking aid
	4	Able to walk without walking aid, but most hold a handrail on stairs
	5	Moderate to severe walking imbalance but able to perform stairs without handrail
	6	Mild imbalance when standing OR walking
	7	Normal walking
**Upper extremity sensory subscore (/3)**	0	Complete loss of hand sensation
	1	Severe loss of hand sensation
	2	Mild loss of hand sensation
	3	Normal hand sensation
**Urinary function subscore (/3)**	0	Inability to urinate voluntarily (requiring catheterization)
	1	Frequent urinary incontinence (more than once per month)
	2	Urinary urgency OR occasional stress incontinence (less than once per month)
	3	Normal urinary function

**Table 2:** Patients’ general data and clinical characteristics.

General Data	*n* = 51 (%)
**Age**	Mean 58.1 years old (max 86, min 27)
**Sex (male/female)**	Male: 30 (59%); Female: 21 (41%)
**Diagnosis**	
**Cervical herniated disk**	11 (21%)
**Cervical stenosis**	36 (71%)
**Cervical herniated disk and stenosis**	4 (8%)
**Previous cervical spine surgery**	5 (9%)
**History of cervical trauma**	2 (4%)
**Instability**	10 (20%)
**Levels of myelopathy**	
**One**	26 (51%)
**Two**	24 (47%)
**Three**	1 (2%)
**Surgical treatment**	
**Anterior**	34 (67%)
**ACDF**	32 (63%)
**Corpectomy**	2 (4%)
**Posterior**	13 (25%)
**Laminectomy**	7 (13%)
**Posterior Arthrodesis**	6 (12%)
**Combined approach**	4 (8%)
**Preoperative mJOA score**	
**Mild**	38 (74%)
**Moderate**	9 (18%)
**Severe**	4 (8%)
**Outcome**	
**Complete recovery**	33 (65%)
**Non complete recovery:**	18 (35%)
**Clinical improvement**	15 (29%)
**Clinical stability**	3 (6%)

**Table 3:** Results of univariate analyses (Chi-square test).

		Complete Recovery		*p*-Value
		**Yes**	**None**	
**Preoperative mJOA score**	Mild	32 (62.7%)	6 (11.8%)	<0.001
	Moderate/Severe	1 (2%)	12 (23.5%)	
**Age**	*n*	33	18	<0.001
	Mean	53.33	66.78	
**ACDF**	Yes	26 (51%)	6 (11.8%)	0.002
	None	7 (13.7%)	12 (23.5%)	

**Table 4:** Results of multivariate regression analysis.

	*p*-Value	OR	CI_95%_
**Age**	0.089	1.123	0.983–1.282
**ACDF**	0.105	7.473	0.661–84.453
**Preoperative mild mJOA score**	0.002	240.6	6.815–8496.220

## Data Availability

The data associated with the paper are available from the corresponding author upon reasonable request.

## Abbreviations

The following abbreviations are used in this manuscript:

DCM	Degenerative Cervical Myelopathy
QoL	Quality of Life
mJOA	modified Japanese Orthopedic Association
SF-36	Short Form-36
ACDF	Anterior Cervical Discectomy and Fusion
SCC	Spinal Cord Compression
MRI	Magnetic Resonance Imaging
CT	Computer Tomography
OPLL	Ossification of the Posterior Longitudinal Ligament
